# Mechanical Performance of CAD/CAM-Milled Versus 3D-Printed Resins for Prosthetic Applications: A Systematic Review and Meta-Analysis

**DOI:** 10.3390/polym18101257

**Published:** 2026-05-21

**Authors:** Carlos Carpio-Cevallos, Luis Chauca-Bajaña, Andrea Ordoñez-Balladares, Benjamín José Martín-Biedma, Byron Velasquez Ron, José Martín-Cruces

**Affiliations:** 1 Dental Sciences, College Dentistry, University of Guayaquil, Guayaquil 090514, Ecuador; carlos.carpioce@ug.edu.ec; 2 Universidade de Santiago de Compostela, Santiago de Compostela, 15705 A Coruña, Spain; 3 School of Dentistry, Universidad Católica de Santiago de Guayaquil (UCSG), Guayaquil 090101, Ecuador; 4 College Dentistry, Universidad Bolivariana del Ecuador, Durán 092406, Ecuador; 5 Oral Sciences Research Group, Endodontics and Restorative Dentistry Unit, School of Medicine and Dentistry, Universidade de Santiago de Compostela, Health Research Institute of Santiago (IDIS), Rúa Entrerríos 1, 15705 Santiago de Compostela, Spain; benjamin.martin@usc.es; 6 Carrera de Odontología, Department Prosthesis Research, Universidad de Las Américas Ecuador (UDLA), Quito 170102, Ecuador; byron.velasquez@udla.edu.ec; 7 Endodontics and Restorative Dentistry Unit, School of Medicine and Dentistry, Universidade de Santiago de Compostela, Rúa Entrerríos 1, 15705 Santiago de Compostela, Spain; pepe3214@gmail.com

**Keywords:** dental materials, computer-aided design, computer-aided manufacturing, 3D

## Abstract

**Background:** Digital fabrication techniques such as CAD/CAM milling and 3D printing are widely used for provisional dental restorations. However, differences in mechanical performance remain controversial. **Objective:** To compare the hardness and flexural strength of CAD/CAM-milled resins versus 3D-printed resins used in restorative dentistry. **Methods:** A systematic review and meta-analysis were conducted following PRISMA 2020 guidelines and registered in PROSPERO (CRD420251045547). Electronic searches were performed in PubMed, Scopus, Web of Science, Embase, and LILACS. In vitro studies comparing CAD/CAM-milled and 3D-printed resins in terms of hardness and/or flexural strength were included. A random-effects inverse-variance model was applied using standardized mean difference (SMD) with 95% confidence intervals (CI). Risk of bias was assessed using the RoB-Iv tool. **Results:** Four studies (n = 124 specimens) were included in the hardness meta-analysis. CAD/CAM-milled resins showed significantly higher hardness (SMD = 2.92; 95% CI: 0.34–5.49; *p* = 0.026), although heterogeneity was high (I^2^ = 94.9%). Funnel plot asymmetry suggested possible small-study effects. For flexural strength, three studies (n = 40 specimens) were analyzed, demonstrating a significant effect favoring milled resins (SMD = 1.28; 95% CI: 0.42–2.14; *p* = 0.0036) with low-to-moderate heterogeneity (I^2^ = 27.8%). Sensitivity analyses confirmed robustness for both outcomes. Overall methodological quality was acceptable, with no high risk of bias identified in strength studies. **Conclusions:** CAD/CAM-milled resins tend to demonstrate higher hardness and flexural strength compared with 3D-printed resins. However, the substantial heterogeneity observed, particularly for hardness, and the potential influence of methodological variability, warrant cautious interpretation of these findings.

## 1. Introduction

Restorative dentistry has undergone a true revolution in recent decades, driven by technological advances that have redefined the way dental restorations are designed and manufactured. One of the most relevant milestones has been the incorporation of CAD/CAM (Computer-Aided Design/Computer-Aided Manufacturing) systems, which allow digital scanning of the oral cavity, the design of customized restorations in specialized software, and the precise fabrication of pieces through milling [1].

In parallel, 3D printing has gained prominence as a versatile and efficient tool. Through additive manufacturing processes such as stereolithography (SLA) and digital light processing (DLP), it is possible to create three-dimensional structures layer by layer from liquid resins that polymerize under ultraviolet light [2,3]. This innovation has expanded production possibilities in both laboratory and clinical settings.

Both methods have demonstrated multiple benefits in daily practice. They have not only significantly reduced treatment time and the number of required visits but have also improved the accuracy of fit, decreased technical errors, and facilitated digital storage of working models [4,5]. CAD/CAM-milled restorations, in particular, offer consistent quality, improved predictability, and reduced risk of cross-contamination, although their implementation may represent a challenge for some professionals due to cost or the associated learning curve [6].

The evolution of materials has been key to supporting these technological advances. In the 1930s, the first methacrylate resins were used as a base for dentures, cured by heat. Decades later, new formulations emerged, such as epoxy resins and subsequently the dimethacrylate BIS-GMA, introduced by Dr. Bowen in the 1960s, which remains one of the most widely used monomers today [7].

Currently, the variety of available polymers allows processing by both milling and 3D printing. Materials such as acrylonitrile butadiene styrene (ABS), polylactic acid (PLA), polyamide (PA), polycarbonate (PC), and thermoset epoxy resins have proven useful for manufacturing everything from crowns to study models [8]. The latter, in particular, require thermal or UV curing to reach their final properties, beginning with low viscosity that progressively increases during polymerization [9].

Thanks to their versatility, these materials can accurately reproduce morphology, occlusion, and contact points of dental structures [10]. In addition, the possibility of direct photopolymerization has simplified processes, avoiding steps such as firing or casting [8].

Among the mechanical properties that determine the clinical success of a restoration, surface hardness and flexural strength stand out [11]. The latter enables the material to withstand masticatory forces without fracturing and depends on both the chemical composition of the material and its processing and curing methods [12,13]. When this resistance is insufficient, fractures may occur, particularly in provisional restorations, compromising both the function and esthetics of the treatment [12].

More recent hybrid materials, due to the incorporation of a resin matrix, have achieved flexural strength similar to that of natural teeth, making them suitable for areas subjected to functional loads [14]. However, wear resistance remains an important clinical challenge, as it directly influences restoration longevity [15]. Details such as polishing, for example, impact surface smoothness and wear resistance, thus affecting morphology over time [16].

Provisional restorations, in addition to fulfilling an esthetic and functional role during treatment, allow the clinician to evaluate and adjust critical aspects such as margins, occlusion, and possible interferences before fabricating the definitive restoration [17]. They may be used in procedures such as immediate implant loading, crown lengthening, or full-arch rehabilitations [18]. Their clinical success depends, among other factors, on proper marginal adaptation, since poor sealing may promote plaque accumulation, cause gingival inflammation, or even lead to pulpal lesions [19].

Comparative studies have shown that, when evaluating marginal and internal fit, crowns milled using CAD/CAM technology tend to exhibit superior physical properties compared with those produced by 3D printing [20]. CAD/CAM-milled resins have overcome many of these limitations by reducing polymerization shrinkage and ensuring a more homogeneous and stable surface over time [21]. This technological evolution has enabled progress toward metal-free restorations that are more esthetic and biocompatible, aligned with current patient demands [22].

The objective of this study was to evaluate the mechanical properties—hardness and flexural strength—of printed resins compared with CAD/CAM-milled resins used in restorative dentistry.

The null hypothesis of this study was that there are no significant differences in mechanical properties between CAD/CAM-milled and 3D-printed resins.

## 2. Materials and Methods

### 2.1. Protocol and Registration

**Figure 1:**
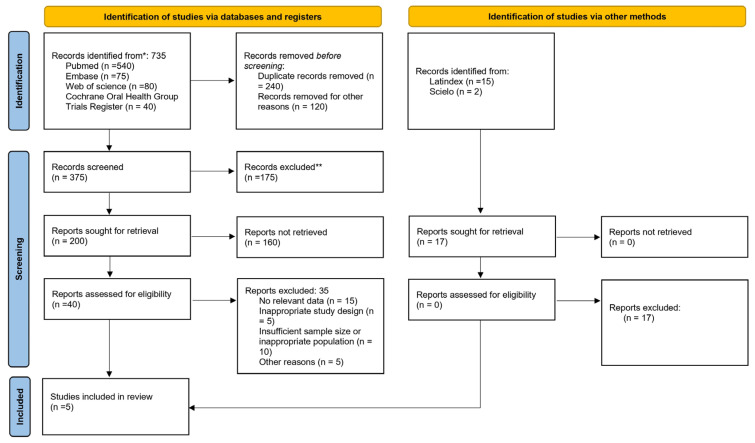
Flowchart of selected studies. * Records identified through database searching and other sources. ** Records excluded after title and abstract screening because they did not meet the predefined inclusion criteria.

A systematic review was conducted following the principles defined in the Preferred Reporting Items for Systematic Review and Meta-Analysis Protocols (PRISMA 2020) (Figure 1) [23].

The review protocol was registered in the International Prospective Register of Systematic Reviews (PROSPERO; Centre for Reviews and Dissemination, University of York), under the identification CRD420251045547.

### 2.2. PICO Question

Do CAD/CAM-milled resins exhibit greater hardness and flexural strength than printed resins in provisional dental restorations?

P (Participants): Articles with in vitro studies using dental resins for provisional restorations were evaluated. I (Intervention): CAD/CAM-milled resins. C (Comparison): Resins obtained through 3D printing techniques (such as SLA or DLP). O (Outcome): The mechanical properties of flexural strength and hardness were compared.

### 2.3. Information Sources and Search Strategy

An electronic bibliographic search strategy was designed and implemented on 3 May 2025. This search included five main databases: PubMed (MEDLINE), Embase, Scopus, Web of Science, and LILACS (Latin American and Caribbean Health Sciences Literature). Additionally, a complementary search of gray literature was conducted using Google Scholar and ProQuest Dissertations & Theses Global. To organize references, manage the bibliography, and remove duplicates, Zotero software (version 6.0.37), developed by the Corporation for Digital Scholarship and the Roy Rosenzweig Center for History and New Media, George Mason University, Virginia, USA, was used. The selection of eligible articles was performed using Rayyan QCRI (Qatar Computing Research Institute, Doha, Qatar), which facilitated independent evaluation and screening by reviewers. The search strategy was based on the combination of keywords and specific MeSH terms adapted to each database, using the Boolean operators AND and OR. Keywords included terms related to the central topic, such as: “dental resins,” “CAD/CAM resins,” “printed resins,” “mechanical properties,” “hardness,” “flexural strength,” and “provisional restorations.” This process was complemented by a manual search in specialized peer-reviewed journals relevant to the field of restorative dentistry.

The full search strategy for PubMed (MEDLINE) was as follows:

(“dental resin” OR “resin materials” OR “CAD/CAM resin” OR “milled resin” OR “printed resin” OR “3D printed resin”) AND (“CAD/CAM” OR “computer-aided design” OR “computer-aided manufacturing” OR “milling”) AND (“3D printing” OR “additive manufacturing” OR “stereolithography” OR “digital light processing”) AND (“mechanical properties” OR “flexural strength” OR “hardness”). No restrictions were applied regarding publication year. Only studies published in English were included. When available, filters were applied to identify in vitro studies. The search strategy was adapted for each database using appropriate controlled vocabulary (e.g., MeSH terms) and syntax.

### 2.4. Inclusion and Exclusion Criteria

The exclusion criteria were as follows: (1) Studies lacking separate quantitative data on the mechanical properties of CAD/CAM resins; (2) studies omitting mention/specification of the types of thermal treatments applied to CAD/CAM; (3) studies involving cemented resin restorations; (4) studies focusing on other conditions, such as cavity preparation designs, surface analysis with tribochemical treatments, and analysis of physical properties; (5) studies that did not include resin materials; (6) studies with duplicated data from another included study; (7) reviews, letters, books, conference proceedings, case–control studies, case reports, case series, opinion articles, technical articles, posters, and guidelines; and full text not available, even after attempting to contact the corresponding authors (three attempts over a period of three weeks).

### 2.5. Study Selection Process and Data Extraction

Two investigators (CC and AO) independently performed data extraction using a customized extraction sheet specifically designed for this review. In case of discrepancies between the two reviewers, these were resolved by a third investigator (BVR), who acted as a blinded evaluator unaware of the study hypothesis. For each included study, the following data were recorded: first author, year of publication, country of origin, study type (in vitro), application (denture base or provisional crown), type of resin evaluated (CAD/CAM or printed), specific CAD/CAM and 3D-printed materials used, processing technology (milling or SLA/DLP 3D printing), sample size per group, mechanical properties analyzed (flexural strength and/or hardness), artificial aging protocols (primarily thermocycling), testing method employed (e.g., three-point bending test), and the main quantitative results reported. The main characteristics of the included studies are summarized in Table 1.

The selection process was conducted in two phases. In the first phase, the titles and abstracts of all identified records were assessed to exclude those clearly irrelevant according to the predefined eligibility criteria. In the second phase, the full texts of potentially eligible studies were reviewed. In cases where any data considered essential for the review were unavailable or unclear, attempts were made to contact the corresponding author by email up to three times, with a one-week interval between each attempt, in order to obtain clarification or additional information.

Although dual independent screening and data extraction were performed, inter-rater agreement statistics (e.g., Cohen’s kappa) could not be calculated retrospectively due to the unavailability of individual reviewer decision records.

To improve the transparency and comparability of the included studies, a detailed summary of methodological and material-related variables is presented in Table 2.

### 2.6. Risk of Bias (RoB) Assessment

The methodological quality of the included studies was independently evaluated by two reviewers using the Risk of Bias tool for In Vitro Studies (RoB-Iv). The following domains were assessed: specimen preparation, randomization, blinding, outcome measurement, and statistical analysis/reporting. Each domain was classified as “low risk,” “some concerns,” or “high risk” of bias. Any disagreements were resolved through discussion until a consensus was reached. The results were presented graphically using traffic light plots and summary bar charts to provide a comprehensive overview of the risk of bias across studies and domains.

### 2.7. Statistical Analysis

#### 2.7.1. Qualitative Analysis

A qualitative synthesis was conducted based on the extracted variables (Table 1). All included studies were in vitro investigations evaluating denture base or provisional crown materials fabricated through CAD/CAM milling or SLA/DLP 3D printing. Flexural strength was the predominant outcome, while hardness was assessed in a subset of studies. Artificial aging through thermocycling was reported in only one study. Overall, the extracted data revealed a consistent methodological focus on comparative mechanical performance between subtractive and additive manufacturing techniques prior to quantitative meta-analytic synthesis.

#### 2.7.2. Meta-Analysis

Quantitative synthesis was performed when at least three studies reported comparable outcomes. Separate meta-analyses were conducted for hardness and flexural strength. Hardness values across studies were obtained using different measurement scales, including Vickers hardness (VHN), Shore hardness, and Barcol hardness, which differ in indentation principles, load application, and scale units. Therefore, the standardized mean difference (SMD) with 95% confidence intervals (CI) was used as the summary effect measure to allow comparison across studies using different measurement scales and units. A random-effects model based on the DerSimonian and Laird inverse-variance method was applied to account for anticipated methodological and material heterogeneity, including differences in resin composition, manufacturing platforms, printing orientation, and post-curing protocols.

Statistical heterogeneity was assessed using Cochran’s Q test, the I^2^ statistic, and Tau^2^ (τ^2^). I^2^ values were interpreted as low (<25%), moderate (25–50%), or high (>50%) heterogeneity. Publication bias was evaluated through visual inspection of funnel plots and, when appropriate, by Egger’s regression test, with *p* < 0.05 considered indicative of potential asymmetry. Given the limited number of included studies per outcome, interpretation of funnel plot asymmetry was performed cautiously. Robustness of the pooled estimates was assessed using leave-one-out sensitivity analysis in which the meta-analysis was repeated sequentially, excluding one study at a time to determine the influence of individual studies on the overall effect size and heterogeneity. Additionally, meta-regression analysis was conducted to explore the potential influence of publication year on effect size. All statistical analyses were performed using Review Manager (RevMan 5.4, The Cochrane Collaboration) and R software version 4.3.2 (R Foundation for Statistical Computing, Vienna, Austria) with the meta package.

Subgroup analyses according to printing technology (SLA vs. DLP), hardness testing method (e.g., Vickers, Shore, and Barcol), and artificial aging conditions (thermocycling) were initially planned to explore potential sources of heterogeneity. However, due to the limited number of included studies and insufficient reporting across studies, these subgroup analyses were not feasible.

## 3. Results

It is important to note that the quantitative synthesis was based on a limited number of included studies, which reflects the current scarcity of standardized comparative research in this field. Therefore, the findings of the meta-analysis should be interpreted with caution, particularly for outcomes with high heterogeneity, as the small sample of studies may influence the stability and generalizability of the pooled estimates.

**Figure 2:**
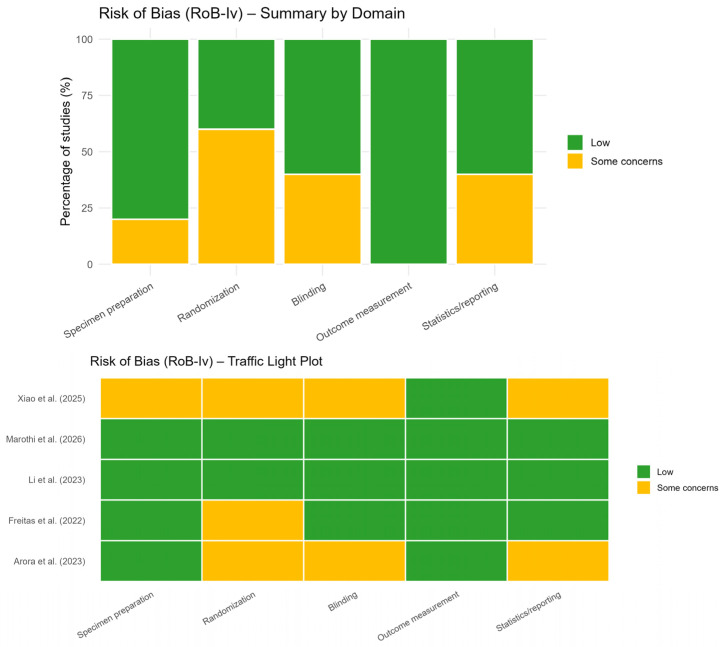
Risk of bias assessment for hardness outcome (traffic light plot and summary plot) [24,25,26,27,28].

The risk of bias assessment using the Risk of Bias tool for In Vitro Studies (RoB-Iv) indicated a generally low to moderate risk of bias across the included studies; however, this finding should be interpreted with caution. Although all studies demonstrated low risk in the outcome measurement domain (100%), suggesting consistent assessment methods, other domains revealed important methodological concerns. Randomization showed the highest level of uncertainty, with approximately 60% of studies classified as having some concerns, indicating potential limitations in allocation procedures. Similarly, blinding and statistical analysis/reporting domains presented moderate concerns in around 40% of the studies, reflecting possible risks of measurement and reporting bias. Importantly, these methodological limitations may not be fully captured by standard risk of bias tools in in vitro research. Variability in specimen preparation protocols, lack of standardized testing conditions, and incomplete reporting of experimental procedures could contribute to inconsistencies in the observed results. Therefore, despite the absence of studies classified as high risk of bias, these factors may partially explain the substantial heterogeneity observed in the meta-analysis, particularly for hardness outcomes (Figure 2).

### Forest Plot of the Meta-Analysis Comparing Hardness Between CAD/CAM Resins and 3D-Printed Resins

**Figure 3:**
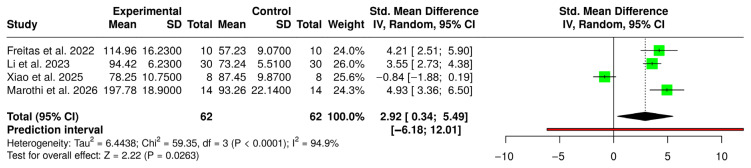
Forest plot of the meta-analysis comparing hardness between CAD/CAM resins and 3D-printed resins [24,25,26,27].

Four studies were included, comprising 62 specimens in the CAD/CAM-milled group and 62 in the 3D-printed group. A random-effects inverse-variance model demonstrated a statistically significant overall effect favoring CAD/CAM-milled resins, with a standardized mean difference (SMD) of 2.92 (95% CI: 0.34 to 5.49; Z = 2.22; *p* = 0.026). Between-study heterogeneity was considerable (τ^2^ = 6.44; χ^2^ = 59.35, df = 3, *p* < 0.00001), with an I^2^ of 94.9%, indicating that most of the observed variability was attributable to true between-study differences rather than sampling error. The prediction interval was wide (−6.18 to 12.01), suggesting substantial uncertainty regarding the magnitude and even the direction of the true effect in future comparable studies. Three studies reported effect sizes favoring milled resins, whereas one study showed a non-significant effect in the opposite direction. Given the high heterogeneity and broad prediction interval, the pooled estimate should be interpreted cautiously. (Figure 3) Given the high heterogeneity observed (I^2^ = 94.9%), the pooled estimates for hardness should be interpreted with caution. The variability across studies may be partially explained by differences in printing technologies (SLA vs. DLP), hardness testing methods, and aging protocols, which could not be formally explored through subgroup analyses due to the limited number of studies.

Visual inspection of the funnel plot revealed marked asymmetry, with most studies distributed on the right side of the pooled effect and limited representation on the left side of the line of no effect. Smaller studies (higher standard errors) tended to report larger effect sizes favoring CAD/CAM-milled resins, suggesting potential small-study effects.

**Figure 4:**
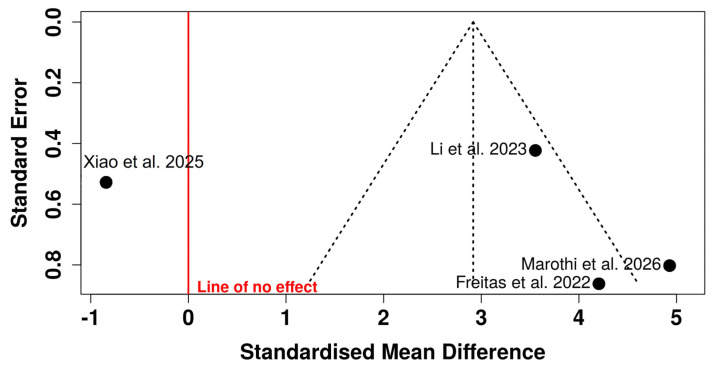
Funnel plot assessing potential publication bias in studies comparing the hardness of CAD/CAM-milled resins and 3D-printed resins [24,25,26,27].

Notably, one study was positioned on the left side of the null line with a relatively higher standard error, further contributing to the asymmetrical distribution. The dispersion of studies outside the pseudo-95% confidence limits reinforces concerns regarding possible publication bias or methodological heterogeneity. Given the small number of included studies (n = 4) and the previously observed high heterogeneity (I^2^ ≈ 95%), the funnel plot should be interpreted cautiously. However, the pattern observed raises the possibility that the pooled effect size may be overestimated due to selective reporting or small-study effects (Figure 4).

**Figure 5:**
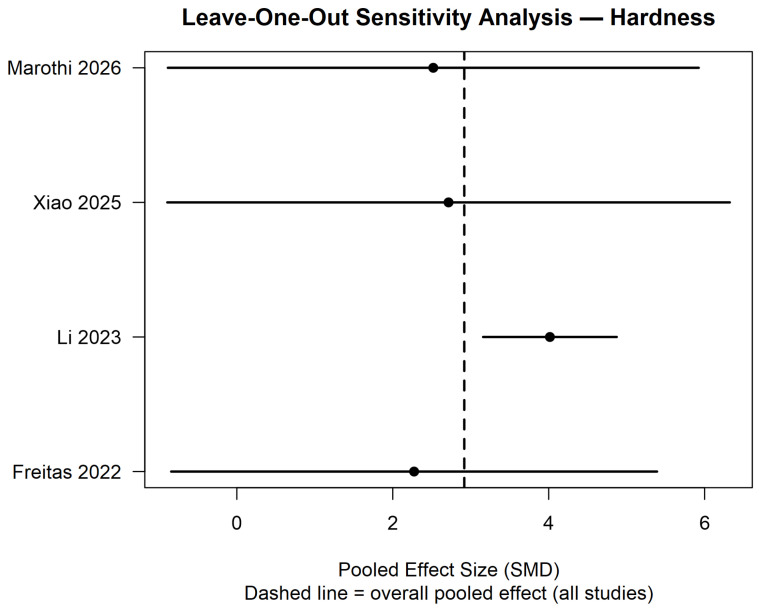
Leave-one-out sensitivity analysis for the hardness outcome [24,25,26,27].

The leave-one-out sensitivity analysis demonstrated that the pooled effect size remained relatively stable after sequential exclusion of each study. In all iterations, the direction of the effect favored CAD/CAM resins, and the confidence intervals largely overlapped with the overall pooled estimate (dashed line). Exclusion of Li 2023 [25] resulted in the greatest shift in the pooled effect size; however, the overall magnitude and direction of the effect were not substantially altered. The removal of Freitas 2022 [24], Xiao 2025 [26], or Marothi 2026 [27] produced minimal changes. These findings indicate that the meta-analytic results for hardness are robust and not driven by any single study (Figure 5).

**Figure 6:**
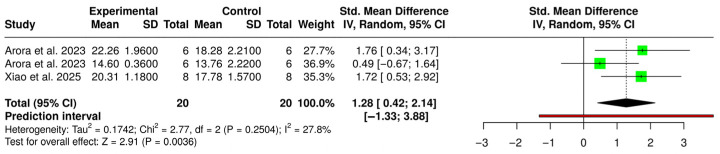
Forest plot of the meta-analysis comparing strength between CAD/CAM resins and 3D-printed resins [26,28].

Three studies were included in the meta-analysis of flexural strength. Sample sizes varied across studies and were analyzed at the group level according to the reported experimental design. Some studies reported combined sample sizes; therefore, all data were standardized prior to inclusion in the meta-analysis to ensure consistency. Using a random-effects inverse-variance model, the pooled standardized mean difference (SMD) was 1.28 (95% CI: 0.42 to 2.14), indicating a statistically significant effect favoring CAD/CAM-milled resins (Z = 2.91; *p* = 0.0036). Between-study heterogeneity was low to moderate (τ^2^ = 0.174; χ^2^ = 2.77, df = 2, *p* = 0.250; I^2^ = 27.8%), suggesting that most of the variability across studies was attributable to sampling error rather than true between-study differences. Study weights were relatively balanced (27.7–36.9%), and all individual effect estimates favored milled resins, although two confidence intervals crossed the null value. The prediction interval was wide (−1.33 to 3.88), reflecting residual uncertainty regarding the magnitude of the true effect in future comparable studies, despite the low statistical heterogeneity observed. Overall, the analysis demonstrates a significant and more consistent advantage of CAD/CAM-milled resins compared with 3D-printed resins for the evaluated mechanical property (Figure 6).

**Figure 7:**
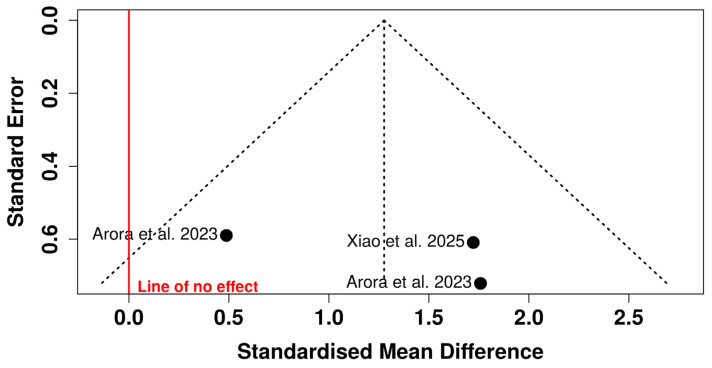
Funnel plot for the detection of publication bias in studies evaluating the flexural strength of CAD/CAM resins versus 3D-printed resins [26,28].

Visual inspection of the funnel plot suggests a relatively symmetrical distribution of studies around the pooled effect estimate. All included studies are positioned on the right side of the line of no effect, indicating consistent directionality favoring CAD/CAM-milled resins. The dispersion of studies appears balanced within the pseudo-95% confidence limits, and no clear clustering of small studies with disproportionately large effects is observed. Although one study lies closer to the null value, its position does not markedly distort the overall symmetry. Given the small number of studies (n = 3), formal interpretation of funnel plot asymmetry is inherently limited. Nonetheless, no strong visual evidence of publication bias or small-study effects is apparent in this analysis (Figure 7).

**Figure 8:**
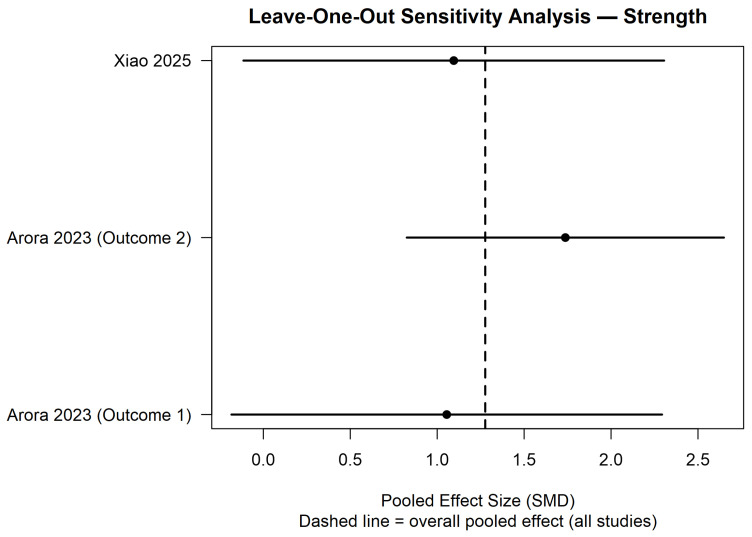
Leave-one-out sensitivity analysis for flexural strength outcome [26,28].

The leave-one-out analysis showed that the pooled effect size remained consistent after sequential exclusion of each study. In all iterations, the effect direction was maintained, and confidence intervals overlapped with the overall pooled estimate (dashed line). Exclusion of Arora 2023 (Outcome 2) [28] produced the largest change in the pooled estimate; however, the magnitude and direction of the effect were not substantially modified. Removal of Arora 2023 (Outcome 1) [28] or Xiao 2025 [26] resulted in minimal variation. These findings indicate that the meta-analytic results for flexural strength are stable and not driven by any single study. (Figure 8).

**Figure 9:**
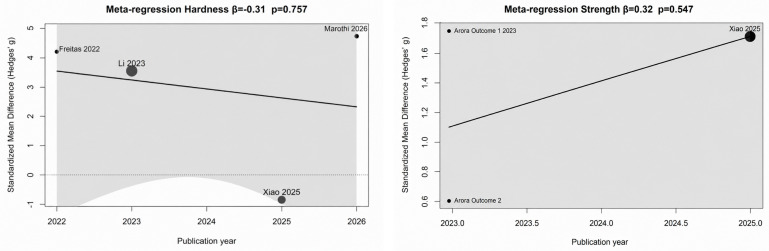
Meta-regression analysis evaluating the influence of publication year on the standardized mean difference in flexural strength between CAD/CAM-milled resins and 3D-printed resins [24,25,26,27,28].

Meta-regression analysis was performed to explore the potential influence of publication year on flexural strength outcomes. A positive but non-significant association was observed (β = 0.32, *p* = 0.547), indicating a slight increase in effect size in more recent studies favoring CAD/CAM-milled resins; however, publication year did not significantly account for the observed variability among studies. Given the limited number of included studies, these findings should be interpreted cautiously (Figure 9).

## 4. Discussion

The present systematic review with meta-analysis comparatively evaluated the mechanical performance of dental resins manufactured through milled CAD/CAM technology and additive manufacturing, with particular emphasis on their application in digital restorative dentistry. The findings demonstrated that milled resins exhibit significantly higher hardness and flexural strength values compared with 3D-printed resins, suggesting that the manufacturing method directly influences the final mechanical stability of these materials. The included experimental studies consistently reported greater structural stability in milled materials. De Freitas et al. [24] observed that CAD/CAM materials present greater homogeneity and mechanical resistance than printed systems, which showed increased susceptibility to degradation following artificial aging. Similarly, Xiao et al. [26] demonstrated that thermocycling has a greater impact on printed polymers, progressively reducing their mechanical properties and suggesting increased vulnerability to hydrolytic degradation.

These differences may be explained by the microstructure resulting from the polymerization process. CAD/CAM blocks are industrially polymerized under controlled pressure and temperature conditions, promoting polymer networks with higher crosslinking density and lower residual monomer content. Vincze et al. [29] reported that such industrial polymerization produces materials with more consistent and predictable mechanical properties compared with those obtained through additive manufacturing techniques. In contrast, printed resins rely on layer-by-layer photopolymerization processes, in which post-curing efficiency and manufacturing parameters directly influence final structural integrity.

The degree of conversion and post-polymerization protocols represent critical factors affecting the mechanical performance of printed resins. Aktug Karademir et al. [30] demonstrated that optimized post-curing significantly increases microhardness and structural stability, whereas Qiu et al. [31] showed that wavelength, light intensity, and exposure time directly influence flexural strength and degree of conversion. These technological variables help explain the greater mechanical variability observed in printed materials and contribute to the heterogeneity detected in hardness analysis.

Printing orientation constitutes another relevant factor. Al-Dulaijan et al. [32] reported significant variations in flexural strength depending on printing direction and post-curing duration, which has been attributed to the inherent anisotropy of additive manufacturing processes. Subsequent investigations have indicated that interlayer interfaces generated during printing may act as stress concentration zones, facilitating crack propagation under functional loading [33,34].

The findings of the present meta-analysis are consistent with recent evidence. Azab et al. [35], in a systematic review with meta-analysis, reported higher flexural strength values in milled materials compared with printed ones, although their analysis primarily focused on denture base materials. Within the restorative context, Tayeb et al. [36] and Park et al. [37] have also described greater mechanical stability in milled materials intended for crowns and definitive restorations, supporting the trend observed in the present study.

Additional physicochemical factors may also influence long-term mechanical performance. Water absorption and hydrolytic degradation have been shown to more severely affect polymers with lower crosslinking density, promoting organic matrix plasticization and progressive reduction in mechanical properties [38]. In contrast, highly polymerized CAD/CAM materials demonstrate greater dimensional stability and resistance to degradation [39], which may explain their more consistent behavior under simulated intraoral conditions.

From a laboratory-based perspective, these findings suggest that CAD/CAM-milled resins may exhibit greater mechanical predictability under controlled in vitro conditions. Printed resins, in turn, may demonstrate comparatively lower mechanical performance in similar experimental settings. However, these results should be interpreted with caution, as all included studies were conducted under in vitro conditions that do not fully replicate the complex oral environment. Therefore, direct extrapolation to clinical indications remains limited. Nevertheless, the development of novel photopolymerizable formulations and improvements in printing and post-curing protocols continue to enhance the mechanical properties of these materials [40,41], potentially reducing the gap currently observed between both technologies.

The high heterogeneity identified in hardness analysis likely reflects differences in composition, filler content, printing technologies, and post-curing protocols among the included studies. Surface hardness is particularly sensitive to the degree of conversion and final polymerization conditions, which explains the observed dispersion. Conversely, the lower heterogeneity observed in flexural strength suggests that this property primarily depends on the overall structural integrity of the material.

Among the limitations of the present study are the limited number of available investigations, the predominance of in vitro studies, and the variability in experimental protocols [42]. The absence of longitudinal clinical studies restricts direct extrapolation to real intraoral conditions, particularly regarding fatigue behavior and long-term mechanical stability.

Overall, the available evidence indicates that the manufacturing method remains a key determinant of the mechanical performance of resins used in digital dentistry. Although milled CAD/CAM resins currently demonstrate more consistent and predictable properties, ongoing optimization of photopolymerizable systems and additive manufacturing processes may progressively reduce the mechanical differences observed between milled and printed materials.

From a polymer science perspective, the apparently higher mechanical performance of CAD/CAM-milled resins under in vitro conditions may be attributed to fundamental differences in polymer network structure and processing conditions. Industrially polymerized CAD/CAM blocks are manufactured under high pressure and temperature, promoting a higher cross-linking density and a more homogeneous polymer network with reduced porosity. This results in improved load distribution and enhanced resistance to crack initiation and propagation. In contrast, 3D-printed resins undergo layer-by-layer photopolymerization, which may lead to incomplete polymerization, lower cross-linking density, and higher residual monomer content, particularly when post-curing protocols are suboptimal.

Additionally, the additive manufacturing process inherently introduces interfacial boundaries between layers, which can act as stress concentration sites and weaken interlayer bonding. These interfaces may facilitate crack propagation under mechanical loading, contributing to comparatively lower mechanical performance. Variations in light exposure, curing depth, and polymerization kinetics across layers may further increase structural anisotropy in printed materials. Collectively, these polymer-level differences provide a mechanistic explanation for the observed variability in mechanical performance between 3D-printed and CAD/CAM-milled resins.

The substantial heterogeneity observed in the hardness meta-analysis may be attributed to multiple methodological and material-related factors. Variations in resin composition, filler content, and degree of conversion across different commercial systems likely contributed to inconsistencies in mechanical performance. In addition, differences in additive manufacturing parameters, including printing orientation, layer thickness, and post-curing protocols, may have further influenced the results. The inclusion of studies evaluating both denture base materials and provisional crowns introduces additional variability due to differences in functional requirements and testing conditions. Furthermore, the relatively small sample sizes and limited number of included studies may have amplified the observed heterogeneity. Variability in testing methodologies may also be related to differences in adherence to international standards, such as ISO 20795-1, ISO 10477, and ISO 4049, which could further affect the comparability of results across studies. These factors should be carefully considered when interpreting the pooled estimates.

This study presents several important limitations that should be considered when interpreting the findings. First, all included studies were conducted under in vitro conditions, which do not fully replicate the complex mechanical, thermal, and biological environment of the oral cavity. Therefore, the direct clinical applicability of these results is limited. Second, the number of included studies was relatively small, which may affect the robustness and generalizability of the meta-analytic estimates. Third, substantial methodological heterogeneity was observed, particularly for hardness outcomes, likely due to differences in resin composition, manufacturing techniques, printing parameters, and post-curing protocols. Fourth, variability in testing methodologies—including differences in specimen preparation, loading conditions, and hardness testing parameters—may further contribute to inconsistencies across studies. Finally, the absence of longitudinal clinical studies prevents the evaluation of long-term performance under real intraoral conditions.

Despite these limitations, the available evidence provides useful insights into the mechanical behavior of resins fabricated using different digital technologies. However, the findings should be interpreted cautiously and within the context of laboratory-based conditions.

Future research should focus on well-designed standardized in vitro protocols to reduce methodological variability and improve comparability across studies. In addition, longitudinal clinical studies are needed to evaluate the long-term mechanical performance and survival of CAD/CAM-milled and 3D-printed resins under real intraoral conditions. Further investigations should also explore the influence of printing parameters, post-curing protocols, and material composition on mechanical properties, as well as the development of novel photopolymerizable materials with enhanced structural stability.

From a clinical perspective, it is important to recognize that the current findings are based on materials and technologies available at the time of the included studies. Given the rapid evolution of 3D printing technologies and the continuous development of new photopolymerizable resin formulations, future materials may exhibit improved mechanical performance that differs from the results observed in the present analysis. Therefore, the current evidence should be interpreted within the context of existing materials, and caution should be exercised when extrapolating these findings to next-generation 3D-printed resins.

Advanced microstructural characterization techniques, such as scanning electron microscopy (SEM) or transmission electron microscopy (TEM), may provide valuable insights into the internal architecture of CAD/CAM-milled and 3D-printed resins. These approaches can reveal differences in layer interfaces, porosity, and fracture patterns, thereby offering direct visual evidence to support the observed mechanical performance disparities. Although such analyses were beyond the scope of the present study, their integration into future research could significantly enhance the understanding of structure–property relationships in these materials.

It should be noted that, although the present review focuses on provisional restorations, a substantial proportion of the included studies evaluated denture base materials. While both applications involve polymer-based systems and share similar manufacturing technologies, their clinical indications differ. Therefore, caution is warranted when extrapolating these findings specifically to provisional crown performance. This represents an inherent limitation of the currently available evidence and highlights the need for more targeted studies focusing exclusively on provisional restorative materials.

Additionally, inter-rater agreement statistics (e.g., Cohen’s kappa) were not calculated, which limits the quantitative assessment of reviewer consistency during study selection and data extraction and represents a methodological limitation of this review.

## 5. Conclusions

This systematic review and meta-analysis demonstrate that CAD/CAM-milled resins exhibit significantly higher hardness and flexural strength than 3D-printed resins for provisional restorations. The advantage was particularly consistent for flexural strength, where heterogeneity was low, and effect estimates were stable. Although hardness also favored milled resins, substantial heterogeneity and potential small-study effects suggest cautious interpretation. Differences in resin composition, manufacturing platforms, printing orientation, and post-curing protocols likely contribute to variability among studies. While CAD/CAM-milled resins currently present more predictable mechanical performance, 3D-printed materials remain a promising alternative that requires further standardization and long-term clinical validation. Future research with standardized methodologies and clinical validation is required to confirm these findings and optimize the performance of emerging additive manufacturing materials.

## Figures and Tables

**Table 1:** Characteristics of the included studies.

Study	Year	Application	CAD/CAM Material	3D-Printed Material	n Per Group	Outcomes Evaluated	Aging Protocol	Main Direction of Results
Freitas et al. [24]	2022	Denture base	CAD-CAM PMMA	3D-printed denture resin	10	Hardness	None	Favors milled
Li et al. [25]	2023	Denture base	CAD-CAM PMMA blocks	SLA/DLP denture resin	30 (combined)	Hardness	None	Favors milled
Xiao et al. [26]	2025	Denture base	CAD-CAM PMMA	3D-printed resin	8	Flexural strength, Hardness	With and without thermocycling	Mixed (FS variable; hardness favors milled)
Marothi et al. [27]	2026	Provisional crown	CAD-CAM provisional composite	3D-printed provisional resin	14 (combined)	Hardness	None	Strongly favors milled
Arora et al. [28]	2023	Denture base	CAD/CAM complete denture resin	3D-printed denture resin	6	Flexural strength	None	Favors milled

**Table 2:** Detailed methodological and material characteristics of the included studies.

Study	Material Type	CAD/CAM Material	3D Printing Method	Printing Orientation	Post-Curing	Aging (Thermocycling)	Hardness Test	Flexural Test	Equipment/Conditions
Freitas et al. [24]	PMMA-based	CAD-CAM PMMA	Not specified	Not specified	Yes	No	Vickers	3-point bending	Standard ISO conditions
Li et al. [25]	PMMA-based	CAD-CAM PMMA blocks	SLA/DLP	Not specified	Not specified	No	Not reported	3-point bending	Universal testing machine
Xiao et al. [26]	PMMA-based	CAD-CAM PMMA	3D printing	Not specified	Yes	Yes	Vickers	3-point bending	Thermocycling included
Marothi et al. [27]	Composite-based	CAD-CAM composite	3D printing	Not specified	Yes	No	Vickers	3-point bending	Standardized conditions
Arora et al. [28]	PMMA-based	CAD/CAM denture resin	3D printing	Not specified	Not specified	No	Vickers (VHN)	Not evaluated	Microhardness tester

## Data Availability

The data to support the findings of this study will be available on request from the corresponding author, C.A.C.C.
